# *Teucrium leucocladum*: An Effective Tool for the Treatment of Hyperglycemia, Hyperlipidemia, and Oxidative Stress in Streptozotocin-Induced Diabetic Rats

**DOI:** 10.1155/2020/3272103

**Published:** 2020-11-12

**Authors:** Najlaa Bassalat, Sibel Taş, Nidal Jaradat

**Affiliations:** ^1^Biology and Biotechnology Department, Faculty of Sciences, Arab American University, Jenin, P.O. Box 240, State of Palestine; ^2^Biology Department, Science Arts Faculty, Uludağ University, Bursa, P.O. Box 16059, Turkey; ^3^Department of Pharmacy, Faculty of Medicine and Health Sciences, An-Najah National University, Nablus, P.O. Box 7, State of Palestine

## Abstract

*Teucrium leucocladum* is among the most used traditional medicinal plants in Palestine, which is used for the treatment of hyperglycemia and colon spasms from ancient times. Therefore, the current investigation aimed for the first time to determine the hypoglycemic, hypolipidemic, and oxidative stress inhibitory effects of the aerial parts (stem and leaves) of *T. leucocladum* hydrophilic (water) extract in streptozotocin- (STZ-) induced diabetic rats (65 mg/kg), given intraperitoneally at a dose of 100 mg/kg for 21 days. The rats were divided into four groups as control (C), control + *T. leucocladum* extract (C + TL), diabetes (D), and diabetes + *T. leucocladum* extract (D + TL). The antioxidant activity was analyzed using in vitro 2,2-diphenyl-1-picrylhydrazyl and in vivo methods by measuring the plasma and tissue malondialdehyde (MDA) levels using a colorimetric assay. On the other hand, glutathione peroxidase (GSH-Px), erythrocyte superoxide dismutase (SOD) enzyme levels, serum paraoxonase (PON), and arylesterase (ARE) enzyme activities were assessed by utilizing standard biochemical kits. Besides, the blood glucose and serum insulin levels were assessed by a glucometer and Rat ELISA Kit, respectively. However, the autoanalyzer was used to evaluate the lipid profile. The diabetic rat group that administered *T. leucocladum* extract showed the best reduction in the tissue and plasma MDA levels and an increase of insulin-releasing potentials. Besides, the serum PON and ARE activities and erythrocyte superoxide dismutase and whole blood glutathione peroxidase enzyme levels were significantly increased in all animals treated with *T. leucocladum* extract. The current investigation demonstrated that *T. leucocladum* manifests antihyperglycemic and antihyperlipidemic effects and also increased the antioxidative defense system and reduced the lipid peroxidation process in experimental diabetic rats.

## 1. Introduction

For thousands of years, people tried to cure their diseases by utilizing various available natural materials and among these were medicinal plants [1, 2]. Recently, it is well recognized that diabetes mellitus is considered one of the most common diseases worldwide, which is characterized as a type of metabolic disorder known by hyperglycemia, hypoinsulinemia, hyperlipidemia, and increased oxidative stress [3]. However, hyperglycemia diminishes pro-oxidant/antioxidant balance by reducing antioxidant levels and raising the standards of free radical [4]. Oxidative stress can cause various pathological complications in diabetic patients, including cardiovascular diseases, cancer, neurological disorders, acute respiratory distress syndrome, and many other diseases [5, 6]. The human body utilizes various antioxidant mechanisms of actions to decrease the damages caused by oxidative stress including enzymatic and nonenzymatic systems [7]. These mechanisms counterbalance the effects of toxic reactive oxygen species (ROS) in human cells and tissues. The enzymatic and nonenzymatic antioxidant compounds include vitamins A, C, and E, catalase (CAT), glutathione reductase (GRx), glutathione (GSH), glutathione peroxidase (GPx), and superoxide dismutase (SOD) [8].

Hypoglycemic oral drugs and insulin injections have many side effects such as syncope, dizziness, nervousness, anxiety, depression, diarrhea, nausea, and vomiting [9]. Therefore, there are increases of interest by diabetic patients and healthcare global systems to search for traditional and natural herbal remedies with antihyperglycemic effect due to their fewer side effects [10].

However, few studies on medicinal plants have investigated their antihyperglycemic, antihyperlipidemic activities, and antioxidative effects in vivo. These plants mainly consist of bioactive secondary metabolic molecules such as polyphenols, vitamins, glycosides, and steroids which have antioxidant, antihyperlipidemic, and antidiabetic effects at the same time [11].

Many investigations have shown that *Teucrium* plant species contain various bioactive compounds such as terpenoids, flavonoids, and iridoids [12]. *Teucrium leucocladum* Boiss. is a very rare aromatic shrubby plant (20–50 cm) with white appressed woolly stem [13]. Previous investigations showed that *Teucrium* plant species contain different types of sesquiterpene and flavonoid contents including cirsimaritin, apigenin 7-glucoside, vicenin-2, apigenin 5-galloyl-glucoside, and luteolin 7-glucoside [14, 15].

To the best of our knowledge, this is the first study investigating the effects of *T*. *leucocladum* plant antihyperglycemic, antihyperlipidemic, and antioxidant potential in STZ-induced diabetic rats. For that, the current experimental work aims to measure the erythrocyte superoxide dismutase (SOD) and whole blood glutathione peroxidase (GSH-Px) levels and serum paraoxonase (PON) and arylesterase (ARE) activities. In addition, it aims to determine the plasma and tissue (*Musculus gastrocnemius*, heart, liver, and kidney) malondialdehyde (MDA) levels to investigate the oxidative status of the rats and also aims to assess the blood lipid profile, including total cholesterol (TC), triglyceride (TG), and high-density lipoprotein-cholesterol (HDL-C) levels.

## 2. Materials and Methods

### 2.1. Plant Materials and Extraction Methods

The aerial parts of *T. leucocladum* (stems and leaves) were collected from the Nablus region of Palestine in May 2017. The plant was identified by pharmacognosist Dr. Nidal Jaradat. The voucher specimen was deposited in the Herbal Products Laboratory at An-Najah National University with a specific code: Pharm-PCT-2411. The collected plant materials were washed several times with distilled water. Afterward, the aerial parts were dried in the shade at a stable normal humidity level and ordinary temperature.

The aqueous extract of *T. leucocladum* was exhaustively extracted by boiling 100 g of plant powder in 1 L distilled water for 2 h at 100°C. The produced extract was then filtered using Whatman filter paper No.1 and dried using a freeze dryer (Mill-Rock Technology, 85bt, Nanjing, China). The obtained dried aqueous extracts were weighed and stored at 4°C until being used.

### 2.2. Animal Models

The current study was conducted with 40 Wistar Albino male rats which were brought from Uludağ University Animal House; their weight average was 200–250 g. The rats were kept in individual cages and were housed in groups of 4 per cage. All the utilized rats were kept in a temperature-controlled environment (25 ± 2°C) on a 12 : 12 h light: dark photoperiod. They were given free access to tap water and basic laboratory food which consists of 25% proteins, 3% vitamins, 35% carbohydrates, and 7% lipids for one week before starting the trial. Experimental protocols, ethical procedures, and policies were authorized according to the Animal Care and Use Committee of Uludağ University (2018-04/13).

### 2.3. Induction of Diabetes

The rats were inducted with diabetes by using single intraperitoneal injections of STZ (65 mg/kg) (BioShop, Ontario, Canada) which was infused with a freshly prepared solution of sodium citrate buffer with pH 4.5, while control rats (C) were received an injection of citrate buffer solution only. The blood glucose levels were measured after 48 h of STZ inductions.

The rats which had blood glucose levels more than 200 mg/dl were considered diabetic and were included in the conducted study. Blood glucose concentration was measured with a Glucostix strip test in a glucometer (Abbott Glucometer Med. Prod., California, United States). Since streptozotocin injection may result in fatal hypoglycemia related to massive insulin release, the diabetic rats were kept on a 5% glucose solution diet for 24 h after STZ injection to prevent hypoglycemia.

### 2.4. Experimental Design and Sample Collection

The Wistar rats were selected randomly and allotted into 4 groups of 10 rats in each one: Group I consists of normal control rats (C), Group II control rats were given intraperitoneally *T. leucocladum* extract (C + TL), Group III consists of STZ-induced diabetic rats (D), and Group IV diabetic rats were given intraperitoneally *T. leucocladum* extract 100 mg/kg (D + TL).

### 2.5. *T. leucocladum* Treatment

One week after injection of STZ, the aqueous extract of *T. leucocladum* was given intraperitoneally for 21 days at a dose of 100 mg/kg for groups II and IV. The daily food and fluid intake, weekly body weight, and blood glucose levels for all groups were recorded through the experiment [16].

At the end of the experimental stage and after 10–12 h of fasting, the blood samples were obtained from all the studied rat groups under light anesthesia by cardiac puncture. Immediately after the collection of blood samples, the skeletal muscles (*Musculus gastrocnemius*), heart, kidney, and liver tissues were removed, rinsed with a standard normal saline cold solution, dried with gauze, and kept in the refrigerator at −20°C for further use. All the blood samples were drawn in individual additive test tubes which contained EDTA and coated with heparin. However, the whole blood samples and erythrocytes in blood were used for the determination of the activities of antioxidant enzymes. Blood samples were stored at −20°C until they were analyzed.

### 2.6. Determination of Biochemical Parameters

Glucose levels in blood samples obtained from each experimental group by cutting the tail of rats every week of the experiment were estimated using blood Glucostix strips (Abbott-Glucometer, USA). The levels of HDL-C, TG, and TC in serum samples were assessed using standard biochemical apparatus as an autoanalyzer (Abbott, USA). However, the serum insulin level in the serum was evaluated using Rat ELISA kit (Lab science, E-EL-R2466, USA). Moreover, the plasma SOD and GSH-Px levels were estimated by utilizing available commercial kit (YL Biotech, Shanghai), and the PON1 and ARE enzyme activities in serum were estimated using a commercial kit (Rel Assay Diagnostics, Mega Tıp, Gaziantep, Turkey).

The kidney, heart, liver, and muscle tissue MDA levels were determined by the thiobarbituric acid (TBA) method using a spectrophotometer at a wavelength of 532 nm (Beckman Coulter Du 730 UV/Vis, USA) [17]. The plasma MDA concentrations were determined using thiobarbituric acid (TBA) assay using a spectrophotometer at a wavelength of 535 nm [18].

### 2.7. Antioxidant Activity of *T. leucocladum* Extract

Stock solutions at a concentration of 1 mg/ml in methanol were prepared from *T. leucocladum* extract and Trolox (Sigma-Aldrich, Germany). Each one of these stock solutions was diluted with methanol to prepare 12 of the working solutions with the following concentrations: 1, 2, 3, 5, 7, 10, 20, 30, 40, 50, 80, and 100 *μ*g/ml. A freshly prepared DPPH solution (Sigma-Aldrich, Germany) (0.002% w/v) was mixed with methanol and with each of the abovementioned working solutions at a 1 : 1 : 1 ratio. Besides, a negative control solution was prepared by mixing the mentioned DPPH solution with methanol in a 1 : 1 ratio, while Trolox which is a vitamin E analog and considered strong antioxidant reagent was used as a positive control. All of these solutions were incubated at room temperature in a dark cabinet for 30 min. At the end of the incubation period, the optical density of these solutions was determined spectrophotometrically at a wavelength of 517 nm. The antioxidant activity of Trolox and *T. leucocladum* extract was estimated using the following formula:

(1) $$\begin{array}{c} \%\text{inhibition of DPPH activity}=\frac{A-B}{A}\times 100\%, \end{array}$$

where *A* and *B* represent the absorbance of the blank and the extract, respectively.

The antioxidant half-maximal inhibitory concentration (IC_50_) for *T. leucocladum* extract and Trolox and their standard deviations were calculated by using BioData Fit edition 1.02 (data fit for biologist) [19]. The antioxidant activity of *T. leucocladum* at the different concentrations mentioned above was expressed in terms of the antioxidant activity of the Trolox standard. This was determined by using the following equation:

(2) $$\begin{array}{c} \%\text{inhibition according to Trolox}=\frac{\text{Trolox }{\text{IC}}_{50}}{\text{extract }{\text{IC}}_{50}}\times \%100. \end{array}$$

### 2.8. Statistical Analysis

As the data were normally distributed and variables were expressed as mean ± SEM. One-way ANOVA tests were used to investigate statistically significant differences. A level of *p* value <0.05 was accepted as statistically significant. In addition, the determination of *in vitro* antioxidant activity was carried out in triplicate. The obtained results were presented as means ± standard deviation (SD). Statistical analyses were conducted using SPSS version 13.0 for Windows.

## 3. Results

The result of the current study showed that the food and water consumption, blood glucose, and serum TC and TG levels were significantly increased in the D group, while the bodyweight of this group significantly decreased compared with the C group rats. However, there were no differences between the C + TL and C groups, while the food and water intake decreased in the D + TL group in comparison with the D group; otherwise, food and water intake decreased in the C + TL group compared with the D + TL group as presented in Table 1. Moreover, the serum TG levels were significantly decreased in the C + TL group and also food and water consumption, blood glucose, and TC and TG levels were significantly decreased in the D + TL group compared with the D group. Also, in the D group, the blood glucose and serum TG and TC levels were elevated, while the serum insulin and HDL-C levels were significantly decreased in comparison with the C group. Furthermore, high levels of insulin and low levels of blood glucose, TG, and TC were significantly detected in the D + TL group in comparison with the D group. The levels of serum HDL-C did not change between the D and D + TL groups as shown in Table 1.

In addition, Table 2 shows that the plasma GSH-Px level was significantly higher in the D + TL group in comparison with the D group while plasma GSH-Px and SOD levels decreased in the C + TL group compared with the D + TL group. Furthermore, the serum PON and ARE activities were increased dramatically in the C + TL group in comparison with the control one. In diabetic rats, the activities of PON1 and ARE were decreased significantly compared with the C group

**Figure 1:**
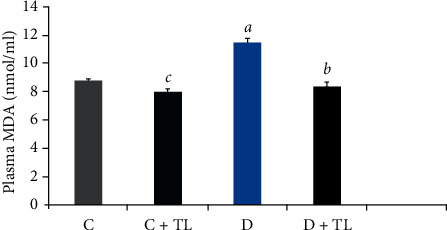
Malondialdehyde (MDA) levels in plasma (nmol/ml) of the control and experimental rats. Data analyzed using one-way ANOVA. Values are expressed as mean ± SEM. ^*a*^*p* < 0.05 between the normal control (C) and diabetic control (D). ^*b*^*p* < 0.05 between the diabetic control (D) and diabetic TL treatment (D + TL). ^*c*^*p* < 0.05 between the normal control (C) and the normal TL treatment (C + TL). ^*d*^*p* < 0.05 between the normal TL treatment (C + TL) and diabetic TL treatment (D + TL).

On the other hand, both the PON1 and ARE activities in the D + TL group were increased significantly in comparison with the D group. Moreover, Figure 1 depicts that the MDA levels did not change in the C and C + TL groups in the kidney and heart tissues, while a significant increase was observed in the MDA levels in the diabetic group. In addition, MDA levels in the heart and kidney tissues were decreased in the C + TL group compared with the D + TL group.

**Figure 2:**
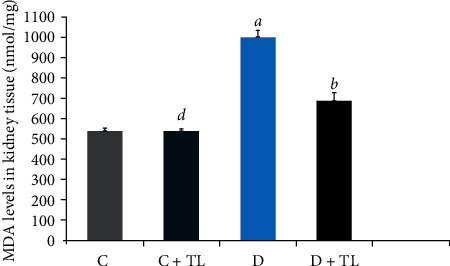
Malondialdehyde (MDA) levels in the kidney tissues (nmol/mg) of the control and experimental rats. Data analyzed using one-way ANOVA. Values are expressed as mean ± SEM. ^*a*^*p* < 0.05 between the normal control (C) and diabetic control (D). ^*b*^*p* < 0.05 between the diabetic control (D) and diabetic TL treatment (D + TL). ^*c*^*p* < 0.05 between the normal control (C) and the normal TL treatment (C + TL). ^*d*^*p* < 0.05 between the normal TL treatment (C + TL) and diabetic TL treatment (D + TL).

**Figure 3:**
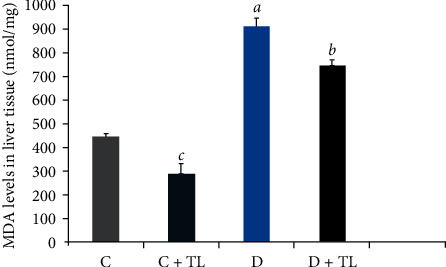
Malondialdehyde (MDA) levels in the liver tissues (nmol/mg) of the control and experimental rats. Data analyzed using one-way ANOVA. Values are expressed as mean ± SEM. ^*a*^*p* < 0.05 between the normal control (C) and diabetic control (D). ^*b*^*p* < 0.05 between the diabetic control (D)and diabetic TL treatment (D + TL). ^*c*^*p* < 0.05 between the normal control (C) and the normal TL treatment (C + TL). ^*d*^*p* < 0.05 between the normal TL treatment (C + TL) and diabetic TL treatment (D + TL).

**Figure 4:**
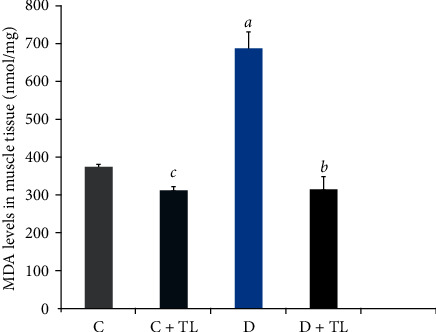
Malondialdehyde (MDA) levels in the muscle tissues (nmol/mg) of the control and experimental rats. Data analyzed using one-way ANOVA. Values are expressed as mean ± SEM. ^*a*^*p* < 0.05 between the normal control (C) and diabetic control (D). ^*b*^*p* < 0.05 between the diabetic control (D) and diabetic TL treatment (D + TL). ^*c*^*p* < 0.05 between the normal control (C) and the normal TL treatment (C + TL). ^*d*^*p* < 0.05 between the normal TL treatment (C + TL) and diabetic TL treatment (D + TL).

**Figure 5:**
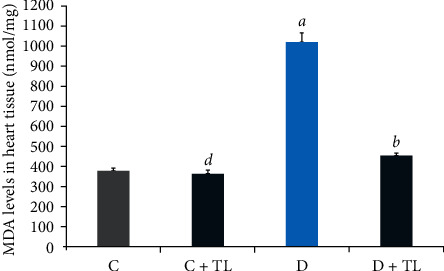
Malondialdehyde (MDA) levels in the heart tissues (nmol/mg) of the control and experimental rats. Data analyzed using one-way ANOVA. Values are expressed as mean ± SEM. ^*a*^*p* < 0.05 between the normal control (C) and diabetic control (D). ^*b*^*p* < 0.05 between the diabetic control (D) and diabetic TL treatment (D + TL). ^*c*^*p* < 0.05 between the normal control (C) and the normal TL treatment (C + TL). ^*d*^*p* < 0.05 between the normal TL treatment (C + TL) and diabetic TL treatment (D + TL).

On the other hand, MDA levels in the blood plasma, heart, skeletal muscle, liver, and kidney tissues were decreased in the D + TL group compared with the D group rats as shown in Figures 1–5.

**Figure 6:**
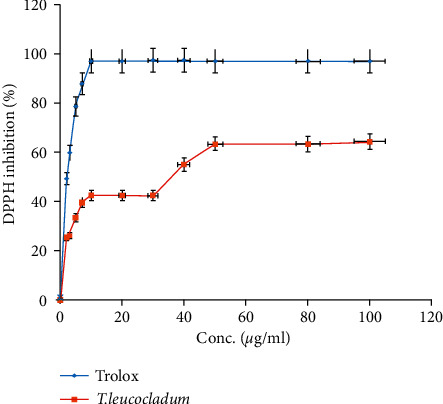
DPPH inhibitory activity by *T. leucocladum* aqueous extract and Trolox. This test was conducted in triplicate and the results were expressed as mean ± SD.

The antioxidant activity of *T. leucocladum* extract was tested by DPPH assay using Trolox as a reference compound. The used concentrations ranged from 1 to 100 *μ*g/ml for the *T. leucocladum* extract as well as for standard Trolox as shown in Figure 6.

The result revealed that the free radical scavenging property was exhibited by *T. leucocladum* extract which has an IC_50_ value of 25.7 *μ*g/ml, while the free radical scavenging property of Trolox was 2.09 *μ*g/ml.

## 4. Discussion

Most of the recently used medicines are initially evaluated on animal models for various purposes. Firstly, it may not be necessary to examine new medicines on humans if preliminary evaluations on animals show that they are not clinically useful. Secondly, studies on animals provide a degree of genetic and environmental manipulation rarely possible in humans. Thirdly, animal studies provide unique insights into the etiology and pathophysiology of the disease and can reveal new targets for the tested medicines. Finally, regulatory authorities concerned with public protection require extensive animal testing to evaluate new medicines for toxicity and to establish safety [20, 21].

Recently, global interests are focusing on the search for organic nutrients, nonnutrient, and traditional medicinal herbs which have antidiabetic and antioxidant potentials. They mainly contain biologically active phytochemical classes such as stilbenes, flavonoids, polyphenols, and carotenoids for reducing the negative impacts of free radicals and oxidative stress damages especially in patients suffering from diabetes mellitus [22, 23].

Hyperglycemia and hyperlipidemia as well as polyuria, polyphagia, and polydipsia are the most prominent symptoms in diabetic patients observed in experimental animals (rats and mice) administration by STZ or alloxan with diabetes mellitus [24]. In this regard, the results of the current study showed that in the diabetic rat group, the food consumption, water intake, blood glucose, and TC and TG levels were significantly increased. At the same time, bodyweight, HDL-C, and serum insulin secretions were decreased in comparison with the C group. The current study showed that *T. leucocladum* extract decreased blood glucose levels and elevated serum insulin levels in the D + TL group. However, one of the active substances of *T. leucocladum* was cirsimaritin; an investigation conducted by Lee et al. showed that it is a biologically active compound that prevented apoptosis in pancreatic beta cells caused by STZ. In this study, the increase of the insulin levels in all groups treated with the plant extract may be caused by the regeneration of the pancreas by cirsimaritin compound [25].

Another one of the biologically active compounds found in *T. leucocladum* is apigenin flavonoid. A study conducted by Park found that the glucose uptake in U937 cells decreased when the cells were processed by apigenin [26].

In our study, the decrease in the blood glucose level in the D + TL group may be caused by the effect of apigenin and other contents presented in the plant. The increase of the insulin levels in parallel with the decrease in blood glucose levels in the D + TL group suggested that this plant has a hypoglycemic therapeutic effect.

Actually, hyperlipidemia is a group of metabolic disorders characterized by hypertriglyceridemia and hypercholesterolemia which is considered the cause of many diseases such as macro-microangiopathy, cardiovascular complications, and metabolic syndrome. Epidemiological studies have shown that the consumption of flavonoid-rich diets, diabetic dyslipidemia (hypercholesterolemia, hypertriglyceridemia, hyperphospho-lipidemia), and coronary heart disease risk can be reduced. In fact, flavonoids act directly by activating enzymes regulating lipid and carbohydrate metabolism in the liver and intestines, by stimulating the fat absorption or by increasing the fat excretion [27].

Agreeing with these facts, the current studied plant species contains as observed in previous investigations luteolin and apigenin flavonoids, which may be the main cause of these observed results. In this study, the decrease in serum TC and serum TG levels in both the C + TL and D + TL groups may have been affected by one or all of the abovementioned biological properties of flavonoids. For that, *T. leucocladum* improves hyperlipidemia in diabetic condition and it is important to show a protective effect in healthy rats.

In the D group, the antioxidant potential has increased because of the deficiency of the antioxidant defense system as well as the increase in the blood glucose and lipid levels. In fact, free radicals may produce damaging effects on the cells, causing lipid peroxidation in the cell membranes such as MDA, which is the last product of lipid peroxidation and one of the oxidative stress biomarkers [28].

The current study has shown a significant increase in MDA levels in blood plasma and tissues (heart, muscle, liver, and kidney) in the D group in comparison with the C group. There was a significant decrease in plasma and tissue MDA levels in the C + TL group (only muscle and liver tissues) and a decrease in the MDA levels in the heart, kidney, liver, muscle, and plasma in the diabetic group given *T. leucocladum* (D + TL) extract compared with the C and D groups. On the other hand, the decrease in MDA levels of the blood plasma, heart, skeletal muscle, and kidney tissues was not observed only in the diabetic group but also in the control group. This is an important point in terms of the protective effect of *T. leucocladum* in healthy individuals.

For human health, it is very important to consume vegetable and fruits or any food supplements containing phenolic molecules which play an essential role in protecting the human organisms against cancer, aging, and inflammations, through the management of oxidative stress [29].

Moreover, in vitro, the antioxidant activity results showed that *T. leucocladum* has a high antioxidant potential compared with Trolox as a reference antioxidant compound. In agreement with our results, many previously conducted studies of *Teucrium* species showed a potential antioxidant effect, for example, extracts derived from *T. polium*, *T. chamaedrys,* and *T. montanum* revealed a significant inhibitory effect with IC_50_ values ranging from 10 to 70 mg/mL [30, 31].

There are enzymatic and nonenzymatic antioxidant defense systems in the human body to prevent the damage caused by reactive oxygen species. SOD and GSH-Px are antioxidant enzymes [32, 33].

Regarding oxidative stress inhibitory activity, it was noticed that the GSH-Px was significantly increased in the D + TL group. In addition, it significantly increased the GSH-Px and SOD activities, while decreased the PON and ARE activities in the D group. At the same time, the PON1 enzyme is involved in the PON enzyme family which is a component of HDL-C. The primary role of PON1 is to protect lipoproteins from the oxidative process. For such a reason, the antioxidant effect of PON1 is usually related with the ability to neutralize hydrogen peroxide and other free radicals. Also, ARE enzyme level is an essential indicator for the PON1 activity. Many conditions can increase oxidative stress such as diabetes, hyperlipidemia, and coronary heart diseases [34]. As shown in this study, the decrease in serum PON1 and ARE activities in diabetic rats may be associated with hyperglycemia, hyperlipidemia, and/or oxidative stress [35].

While the revealed results in the current study showed that the activities of PON1 and ARE enzymes were increased significantly in the C + TL and D + TL groups compared with the C and D groups, respectively. It is important that the PON and ARE activities increased in the C + TL and D + TL groups because this suggests that this plant has a strong antioxidant effect in increasing the synthesis of enzymes in both groups. As previously documented, the decrease in ARE activity in the diabetic condition may be due to oxidative modification of the nucleic material and/or transcription factors or due to the glycation process [36]. Polyphenols increase paraoxonase 1 gene expression by an aryl hydrocarbon receptor-dependent mechanism [37].

To the best of the authors' knowledge, the current study provides the first evidence of the hypoglycemic, hypolipidemic, and antioxidant potential of the current studied traditional medicinal plant.

## 5. Conclusion

The findings of the current study concluded that *T. leucocladum* extract can be used as an antihyperglycemic, antihyperlipidemic, and powerful antioxidant agent as a treatment of diabetes or as a supportive therapy. A literature survey on *T. leucocladum* extract has shown that no biomedical analysis has previously been established on the serum insulin, TC, TG, HDL-C, SOD, GSH-Px, PON, ARE, and MDA potentials. Further, toxicological and clinical experiments are required to evaluate this plant species and its possible applications in the pharmaceutical fields.

## Figures and Tables

**Table 1:** Body weight, food and water consumption, and metabolic parameters of the control and experimental groups of rats.

Group	C	C + TL	D	D + TL
Food intake (g/24 h)	17.4 ± 0.46	18.8 ± 0.56^*d*^	36.6 ± 0.82^*a*^	31.9 ± 1^*b*^
Water intake (mL/24 h)	34.5 ± 1.5	36.1 ± 1.1^*d*^	182.7 ± 3.3^*a*^	61.2 ± 1^*b*^
Final body weight (g)	255.4 ± 3.2	226.4 ± 8	202.7 ± 4^*a*^	226.8 ± 12
Glucose (mg/dl)	126.2 ± 3.4	125.8 ± 1.1	534.2 ± 10.6^*a*^	507.3 ± 10.8^*b*^
Insulin (ng/ml)	1.7 ± 0.23	1.6 ± 0.24	0.5 ± 0.08^*a*^	0.99 ± 0.08^*b*^
TC (mg/dl)	58 ± 1.3	54.8 ± 0.8	89.8 ± 3.7 ^*a*^	54.4 ± 2.2^*b*^
TG (mg/dl)	78 ± 2.3	47.8 ± 2.8^*c*^	328.5 ± 38^*a*^	67.8 ± 3.1^*b*^
HDL-C (mg/dl)	53.6 ± 1.4	55.7 ± 3.2	47.1 ± 2.1^*a*^	55.4 ± 1.7

Values are expressed as mean ± SEM for ten rats in each group. Data analyzed using two-way ANOVA. ^*a*^*p* < 0.05 between the normal control (C) and diabetic control (D). ^*b*^*p* < 0.05 between the diabetic control (D) and diabetic TL treatment (D + TL). ^*c*^*p* < 0.05 between the normal control (C) and the normal TL treatment (C + TL). ^*d*^*p* < 0.05 between the normal TL treatment (C + TL) and diabetic TL treatment (D + TL).

**Table 2:** The GSH-Px, SOD, PON1, and ARE activities in the control and experimental groups of rats.

Group	C	C + TL	D	D + TL
Plasma GSH-Px (ng/mL)	8.5 ± 0.24	9.2 ± 0.31^*d*^	9.9 ± 0.42^*a*^	11.7 ± 0.59^*b*^
Plasma SOD (ng/mL)	0.89 ± 0.1	0.97 ± 0.1^*d*^	1.37 ± 0.04^*a*^	1.39 ± 0.08
PON (U/L)	135.3 ± 8.8	187.9 ± 11.3^*c*^	61.5 ± 2.1^*a*^	137.8 ± 20^*b*^
ARE (U/L)	141.8 ± 1.6	175.3 ± 5.2^*c*^	60.7 ± 2.8 ^*a*^	158.5 ± 9.5^*b*^

Values are expressed as mean ± SEM for ten rats in each group. Data analyzed using two-way ANOVA. ^*a*^*p* < 0.05 between the normal control (C) and diabetic control (D). ^*b*^*p* < 0.05 between the diabetic control (D) and diabetic TL treatment (D + TL). ^*c*^*p* < 0.05 between the normal control (C) and the normal TL treatment (C + TL). ^*d*^*p* < 0.05 between the normal TL treatment (C + TL) and diabetic TL treatment (D + TL).

## Data Availability

The data used to support the findings of this study are available from the corresponding author upon request.
